# Diversity and evolutionary dynamics of universal stress proteins in the *Liquorilactobacillus* genus

**DOI:** 10.1007/s11274-026-04821-4

**Published:** 2026-02-28

**Authors:** Dayane da Silva Santos, Allyson Andrade Mendonça, Nara Suzy Aguiar Freitas, Marcos Antônio De Morais Jr

**Affiliations:** 1 Department of Genetics, Federal University of Pernambuco, Av. Moraes Rego, 1235, Cidade Universitária, Recife, PE 50670-901 Brazil; 2 Department of Biology, Federal Rural University of Pernambuco, Recife, Brazil

**Keywords:** Universal stress protein, Lactic acid bacteria, Stress adaptation, Protein evolution

## Abstract

The genus *Liquorilactobacillus* comprises lactic acid bacteria frequently associated with liquid fermentation environments, where they are exposed to multiple stressors such as pH fluctuations, ethanol accumulation, osmotic pressure, and nutrient limitation. Universal stress proteins (USP) are key molecular components that enable bacterial adaptation and persistence under these challenging conditions. In this study, we investigated the diversity, structural features, and evolutionary dynamics of USP genes across 13 type strains of *Liquorilactobacillus* species. A total of 65 *usp*-encoding genes were identified, ranging from three to six copies per genome, all predicted to be cytoplasmic and exhibiting the conserved Usp fold. Phylogenetic analysis revealed that Usp diversification resulted primarily from ancestral gene duplication events followed by gene losses, with no evidence of recent lineage-specific expansions. Approximately half of the proteins retained a canonical ATP-binding motif, while others lacked it or contained degenerate variants, suggesting an evolutionary trajectory from an ancestral non-ATP-binding form toward a gain-of-function motif. Codon-level selection analysis indicated strong purifying selection acting on most sites, with substitutions concentrated in loop regions that may contribute to functional divergence. A focused analysis of *Liquorilactobacillus vini* revealed structural and sequence heterogeneity among its five Usp paralogs, correlating with differential stress-response expression patterns. Overall, our findings demonstrate that Usp evolution in *Liquorilactobacillus* reflects a balance between conservation and diversification, highlighting their central role in stress adaptation and ecological success in dynamic fermentation environments.

**Supplementary Information:**

The online version contains supplementary material available at 10.1007/s11274-026-04821-4.

## Introduction

The genus *Liquorilactobacillus* comprises 13 species of lactic acid bacteria frequently associated with liquid fermentation environments, including alcoholic beverages, cocoa, tea, and other plant-based substrates (Zheng et al. 2020). Several species, such as *Liquorilactobacillus sicerae* (Puertas et al. 2014), *Liquorilactobacillus nagelii* (Larini et al. 2024), and *Liquorilactobacillus vini* (Rodas et al. 2006), have been reported as dominant members of microbial consortia in fermentations of beverages such as water kefir, kombucha, and wine, underscoring their relevance to both traditional and industrial processes.

One of the most remarkable features of *Liquorilactobacillus* is its capacity to withstand diverse stress conditions that are intrinsic to fermentation environments. During these processes, cells are exposed to fluctuations in pH, ethanol accumulation, osmotic stress, and nutrient limitation (Mendonça et al. 2020). Genomic and functional studies have revealed that *Liquorilactobacillus* species harbor numerous genes encoding universal stress proteins (Usp), which are essential for persistence in dynamic ecosystems and for maintaining metabolic activity under challenging conditions (Mendonça et al. 2020; Santos et al. 2024).

The universal stress protein (USP) superfamily is evolutionarily conserved and widely distributed across bacteria, archaea, plants, fungi, and animals (Hingley-Wilson et al. 2010). The first Usp was identified in *Escherichia coli* in 1992, where it was shown to play a central role in stress adaptation, leading to its designation as a “universal” stress protein (Nyström and Neidhardt 1992). Since then, additional functions have been reported, including roles in phosphorylation and glucose metabolism, biofilm formation, adaptation to carbon, nitrogen, and phosphate starvation, and tolerance to antibiotic exposure (Vollmer et al. 2018). Usp have also been associated with protein stabilization, DNA repair and proper refolding, electron transport, and the activity of flavoproteins (Aravind et al. 2002). Based on structural similarity and amino acid (AA) sequence homology, Usp are classified into two major subclasses. Proteins belonging to the first subclass contain an ATP-binding motif in the C-terminal region (G-2X-G-9X-G(S/T), while the second group lacks ATP-binding residues (Kvint et al. 2003; Vollmer et al. 2018).

In *Liquorilactobacillus*, Usp are expected to play important roles in metabolic flexibility and robustness under stress conditions, supporting their biotechnological application in controlled fermentations, probiotics, and food biotechnology. However, the diversity, distribution, and evolutionary history of Usp within this genus remain largely unexplored. Addressing this knowledge gap is crucial to understanding how these proteins contribute to the ecological success and industrial relevance of *Liquorilactobacillus* in liquid fermentation environments.

## Materials and methods

### Genomic dataset and gene annotation

The genome sequences of nomenclatural type of species from the thirteen *Liquorilactobacillus* species available in the ENA (European Nucleotide Archive) and NCBI (National Center for Biotechnology Information) public databases, only complete assembled genomes and high coverage were selected. Gene annotation was performed by Bakta Web 1.9.1 (Schwengers et al. 2021) at (http://bakta.computational.bio) using default settings. After genome annotation, sequences in FASTA format of nucleotides and amino acids were created for the universal stress proteins of *Liquorilactobacillus*.

### Identification of universal stress protein domain and prediction of subcellular localization

Protein sequences from the studied genomes were analyzed using the HMMER suite to identify homologs of the target protein families. Hidden Markov Models (HMMs) corresponding to the protein families of interest were obtained from multiple sequence alignments of known homologs. The hmmscan or hmmsearch functions were used with default parameters, and hits with an E-value ≤ 1e^− 5^ were considered significant. All retrieved sequences were further validated by checking domain architectures and sequence integrity. The subcellular localization of the identified proteins was predicted using PSORTb (version 3.0) for Gram positive bacteria. Protein sequences were submitted to the PSORTb web server (https://www.psort.org/psortb/*)* The algorithm integrates multiple prediction modules, and only predictions with a high confidence score (≥ 7.5) were considered. The localization categories included cytoplasmic, cytoplasmic membrane, periplasmic, outer membrane, and extracellular.

### Homology modeling

The three-dimensional structures of Usp were predicted using the SWISS-MODEL server (https://www.swissmodel.expasy.org/*).* Amino acid sequences were submitted to the automated modeling pipeline, which performs template identification through sequence similarity searches against the SWISS-MODEL template library (SMTL). The best template for each target was selected based on sequence identity, coverage, and GMQE (Global Model Quality Estimation) scores. Homology models were then generated by comparative modeling using the selected templates. The quality of the resulting models was evaluated using the QMEAN (Qualitative Model Energy Analysis) scoring function provided by the server. Only models with acceptable GMQE and QMEAN values (close to 0) were considered reliable and used for further structural analysis and visualization. Homology between *Liquorilactobacillus* Usp proteins using the five *Li.vini* Usps as a model was inferred based on the genetic distance estimated by the Poisson model, using MEGA 11 software (Tamura et al. 2021). Proteins were considered homologous when they presented divergences less than 0.07 and clustered on the same branch of the phylogenetic tree, updated from previously described approaches for identifying conserved orthologs (Rost 1999; Nei and Kumar 2000; Pearson 2013).

### Multiple sequence alignment and phylogeny

The nucleotide and amino acids sequences of the genes encoding universal stress proteins were aligned using the MUSCLE algorithm implemented in MEGA version 13 (Molecular Evolutionary Genetics Analysis). The resulting multiple sequence alignment was manually inspected to remove poorly aligned regions and gaps. Phylogenetic relationships were inferred using the Maximum Likelihood (ML) method implemented in MEGA 13, applying the Tamura-Nei substitution model as determined to be the best-fit model for the dataset. The robustness of the inferred tree was assessed by bootstrap analysis with 500 replicates. Species tree based on the 16 s gene was also generated in the Mega 13. Newick format trees were added in Notung 2.9 (Chen et al. 2000), a gene tree-species tree reconciliation software package that supports duplication-loss (DL) and duplication-transfer-loss (DTL) event models with a parsimony-based optimization criterion. Program default parameters were used. The timetree was inferred by the RelTime method (Tamuka et al. 2012). For this analysis, the Poisson correction model (Zuckerkand and Pauling 1965) of amino acid substitutions was used. Branch lengths were computed using the Neighbor-joining method.The RelTime analysis incorporated 2 calibration constraints that were used to derive minimum and/or maximum bounds at nodes with constraints. The final tree model was obtained from interactive tree of life (iTOL) v6 (Letunic and Bork 2024).

### Evolutive history of universal stress protein of *Liquorilactobacillus*

The genes encoding the universal stress protein (Usp) in *Liquorilactobacillus* were analyzed using the Datamonkey Adaptive Evolution Server. Coding sequences were aligned at the codon level with MUSCLE, and alignments were manually curated to preserve the reading frame. Selective pressure was estimated under the multiple-hit codon substitution model (Multihit). This model accounts for both synonymous substitutions (codon changes that do not alter the encoded amino acid) and nonsynonymous substitutions (codon changes that result in amino aci replacements). Fixed Effects Likelihood (FEL), for multiple nucleotide changes within the same codon during a single evolutionary event, providing a more accurate estimation of substitution rates. For each codon, the rates of synonymous (dS) and nonsynonymous (dN) substitutions were inferred, and the ratio (dN/dS) was used to classify codons as evolving under purifying selection (dN/dS < 1), neutral evolution (dN/dS ≈ 1), or diversifying selection (dN/dS > 1).

## Results

### Universal Stress protein of *Liquolactobacillus vini*

Mendonça et al. (2020) studied the response of *Li. vini* to different stress condition, which included the expression profile of genes encoding the Universal Stress Proteins (Usp). That was the only report on the *usp* genes in the genus *Liquorilactobacillus* so far. Therefore, the *Li. vini* genes were used as a reference in the present work for the analysis of the evolution of *usp* genes in the genus.

**Fig. 1:**
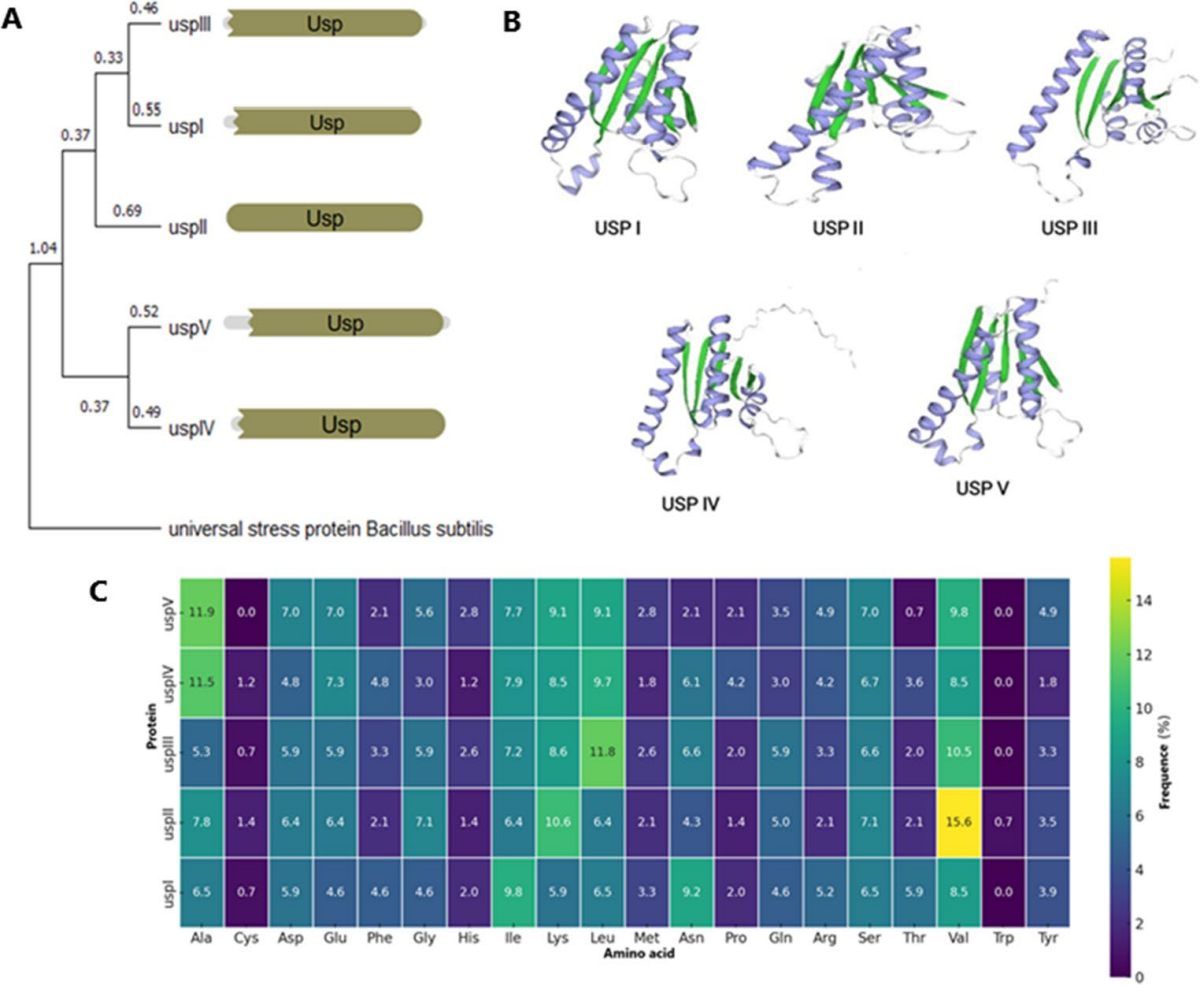
Comparative analysis of universal stress proteins (Usp) in *Liquorilactobacillus vini*. (**A**) Phylogenetic tree of five USP homologs (UspI–UspV) based on sequence similarity, using the *Bacillus subtilis* Usp as an outgroup. Numbers indicate evolutionary distances. (**B**) Predicted 3D structures of the five Usp, highlighting conserved β-sheets (green) and α-helices (purple), showing structural variations among paralogs. (**C**) Heatmap showing the frequency (%) of AA in each Usp protein sequence. Color gradient reflects AA frequency, from low (purple) to high (yellow)

A detailed analysis was performed with the proteins encoded by the five genes in the genome of fuel-ethanol *Li. vini* strain JP 7.8.9 (Fig. 1a). These proteins were grouped into two clusters. The first cluster, composed of UspI, II, and III, represents proteins with the ATP-binding motif, as defined by the bacterial consensus sequence G-2X-G-9X-G-(S/T) (Tkaczuk et al. 2013), where X represents any amino acid. The second cluster comprises Usp IV and V with a degenerated ATP-binding motif. UspII protein contains only the USP domain, while UspI and V also contain N-terminal extra AA residues and UspIII and IV contain both N- and C-terminal extra AA residues (Fig. 1a). All proteins exhibited the canonical tertiary structure containing five α-helices and one barrel formed by five β-sheets structures, albeit with variations in folding (Fig. 1b). The greatest AA divergence was detected in UspII, particularly due to its higher proportion of valine residues (Fig. 1c). Based on this set of information, we analyzed their homologs in the genomes of the other 12 species of the genus.

### Diversity of *usp* genes in *Liquorilactobacillus* genus

Sixty-five genes encoding Universal Stress Proteins were identified in the 13 type-strain genomes. The number of *usp*-coding genes varied from three in *Li. capillatus*, four in *Li. aquaticus* and *Li. cacaonum*, five in *Li. vini*, *Li. hordei*, *Li. mali*, *Li. uvarum* and *Li. sucicola* to six in *Li. sicerae*, *Li. satsumensis*, *Li. hordei*, *Li. ghanensis* and *Li. nagelii*. The strains harbored between three and six *usp* copies among genome sizes. Despite the weak positive correlation, no significance was observed between genome size ranging from 1.9 Mb to 2.7 Mb and the number of *usp* genes in the bacterial genomes (*p* > 0.05). The subcellular localization analysis using PSORTb indicated that all the Usp are cytoplasmic, confirming that all these proteins can be truly considered Universal Stress Protein.

**Fig. 2:**
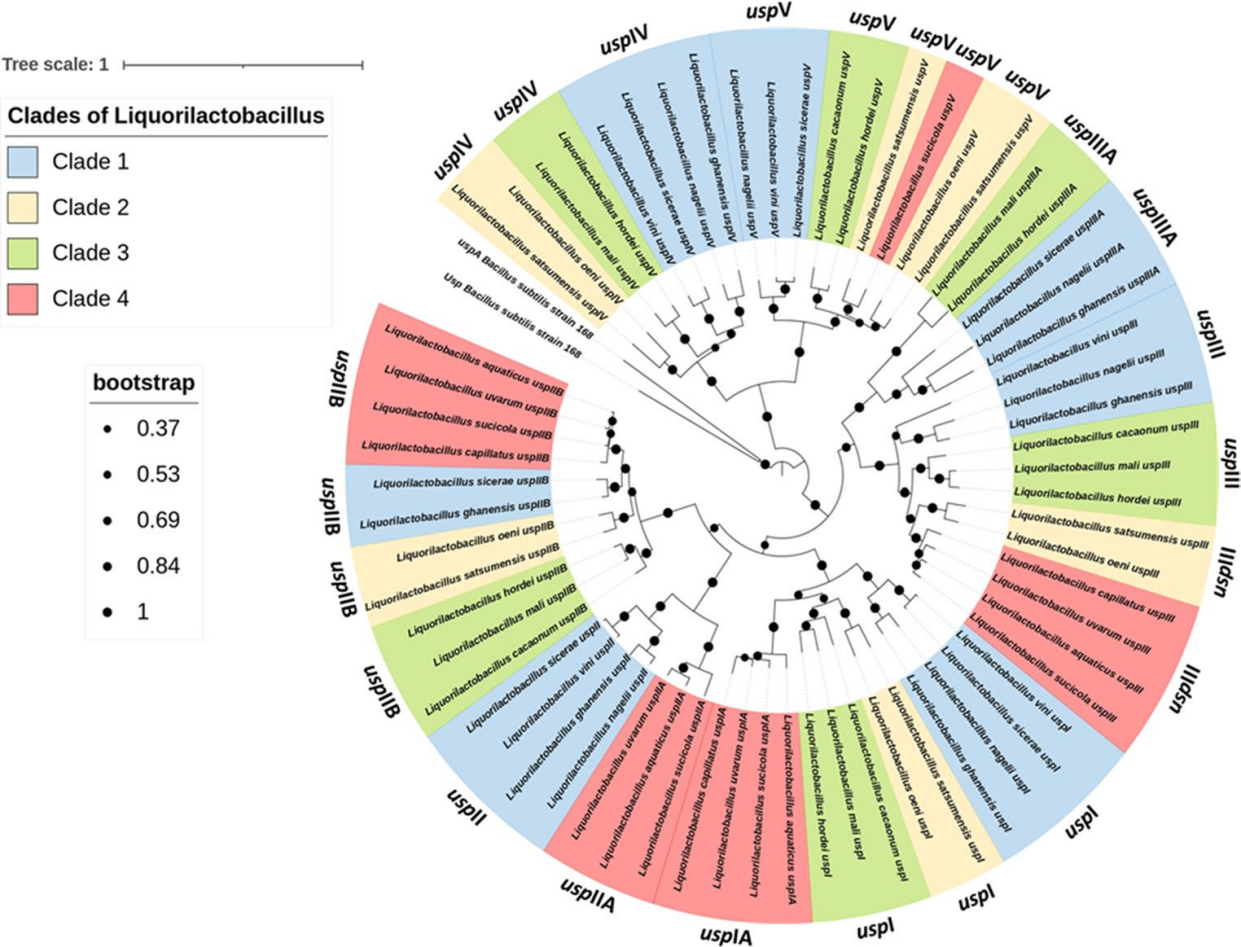
Phylogenetic relationships and correlation analysis in *Liquorilactobacillus*. Maximum likelihood phylogenetic tree of *Liquorilactobacillus* strains, showing four major clades (Clade 1–4) highlighted in different colors. Bootstrap support values are indicated by circle sizes (from 0.33 to 1)

Phylogenetic analysis based on maximum likelihood (Fig. 2) showed that *usp* genes clustered according to the four species clades previously described for this genus (Santos et al. 2024). The tree was rooted with two *usp*A genes from *Bacillus subtilis*, one that encodes a protein that harbors and one that did not harbor the C-terminal ATP-binding motif, since the *Bacillus* group was considered an ancestor of the lactobacilli group (Fig. 2). The gene nomenclature of the other 12 species studied was adjusted according to the degree of homology with each of the five genes of *Li. vini*, which includes the presence or absence of the ATP-binding motif (supplementary material). Each type of Usp (from I to V) was represented in the four clades of species of the genus *Liquorilactobacillus*. The only exception is the absence of proteins homologous to UspIV in clade 4 (Fig. 2). The species *Li. sicerae* and *Li. ghanensis* (from clade 1) have two UspII encoding genes. The species *Li. nagelii* and *Li. ghanensis* (clade 1) and *Li. mali* and *Li. hordei* (clade 3) have two UspIII encoding genes. Finally, *Li. satsumensis* (clade 2) has two UspV encoding genes.

**Fig. 3:**
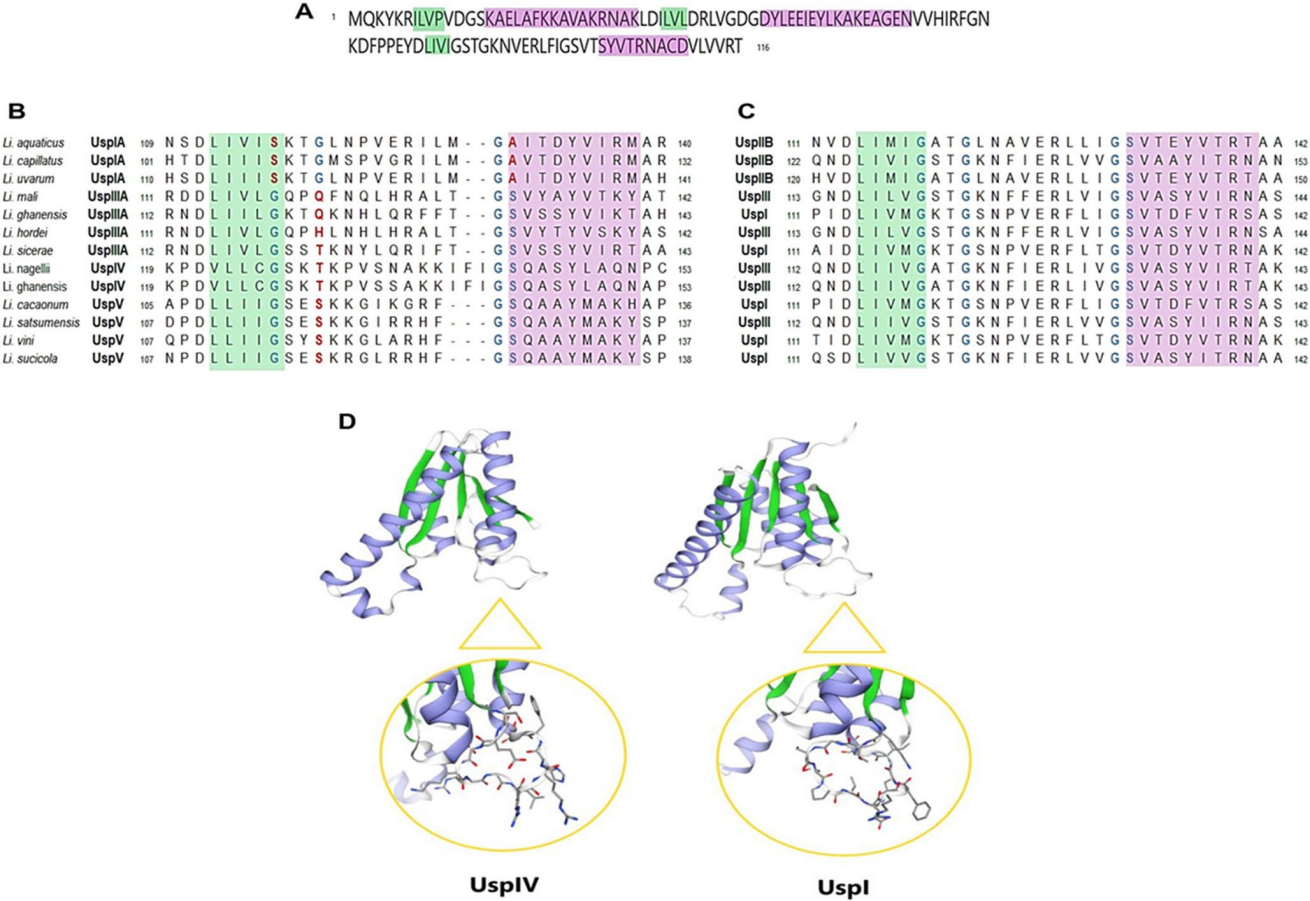
Conserved regions and ATP-binding motif variation in universal stress proteins (Usp) of the *Liquorilactobacillus* genus. (**A**) Consensus amino acid sequence derived from 65 Usp proteins identified across *Liquorilactobacillus* species. (**B**) Multiple sequence alignment of Usp proteins lacking the ATP-binding motif. (**C**) Alignment of Usp proteins containing the ATP-binding motif (G–X–TG–9X–GS) in the C-terminal region. (**D**) Predicted 3D models showing the overall protein fold and the region corresponding to the ATP-binding site (magnified). The USP without the motif (UspIV) lacks the characteristic glycine-rich loop present in the ATP-binding Usp (UspI)

The coding sequences of these 65 genes revealed the production of proteins with lengths ranging from 139 to 186 AA, all containing the canonical five α-helices and five β-barrel structures and one USP consensus sequence of 116 AA (Fig. 3A). Protein alignment revealed that Usp can be classified into two subclasses based on the absence (45%) (Fig. 3B) and the presence (55%) (Fig. 3C) of an ATP-binding motif. In the second subclass, this motif was located in the C-terminal region of the proteins, forming a pocket for ATP/GDP binding between an α- and β-sheets. Moreover, this motif showed a signature of G-X-TG-9X-GS, which seemed to be specific for the *Liquorilactobacillus* genus. Protein modeling of the consensus sequence of the two subclasses revealed that the substitution of the first and/or second glycine residue and the substitution of the serine residue in the ATP-binding motif signature resulted in a structural alteration in the pocket that serves for the binding of the ATP molecule (Fig. 3D), inactivating this pocket.

**Fig. 4:**
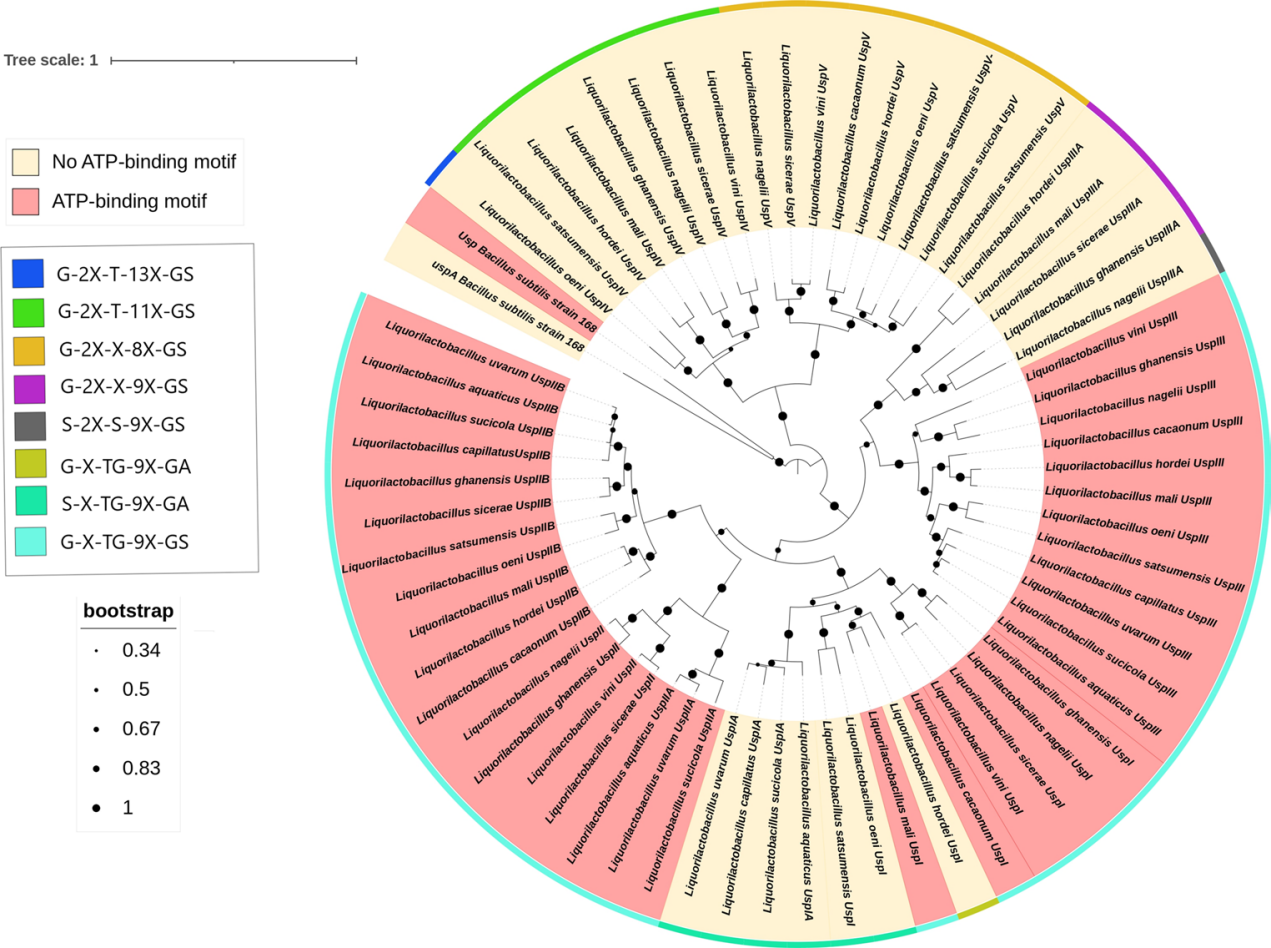
Phylogenetic tree of universal stress proteins (USPs) from *Liquorilactobacillus* species. Proteins are grouped according to the presence (red) or absence (yellow) of the ATP-binding motif. The outer colored ring represents A-loop motif variants. Circle sizes indicate bootstrap support values

The phylogenetic analysis of protein sequences based on maximum likelihood revealed that proteins lacking the ATP-binding motif represented the basal branches (Fig. 4). Therefore, the presence of both the ATP-binding site and this conserved loop within a single, well-supported clade suggests a single evolutionary gain of the ATP-binding capability in a common ancestor, followed by stabilizing selection maintaining the A/P-loop structure.

The so-called degenerate motif displayed several distinct A-loop variants, with different amino acid signatures (Fig. 4). The most basal protein cluster consisted only of species from the basal clades 1 and 2, presenting the signature G-2X-T-11X-GS, which represents the most basal amino acid sequences in the A/P-loop structure in the *Liquorilactobacillus* genus (Fig. 4). On the other hand, the second most basal cluster already includes species representing all four clades. According to protein phylogeny, the third cluster, which has the signature G-2X-X-9X-GS, was formed from the common ancestor that gave rise to the signature that ultimately led to the ATP binding motif, since the only difference between them is the position occupied by the second glycine. The term “degenerate ATP-binding motif” is common in the literature and suggests that the motif existed and was modified throughout evolution. Given that the absence of this motif appears to be a plesiomorphic trait (Fig. 4), we will use the term “gain of function” as the most likely evolutionary path for this trait. Interestingly, proteins that lack this ATP-binding motif reappeared in the central branches of the phylogenetic tree, indicating atavism, that is, a return to the ancestral characteristic (Fig. 4).

Phylogenetic analysis of *Liquorilactobacillus* Usp sequences with divergence time (million years) revealed two major divergence events within the *Liquorilactobacillus* lineage (Figure S1). The root of the tree, dated at 56.2 million years ago (MA), represents the ancestral origin of the *usp* gene after the split from the outgroup (*Bacillus subtilis*). The second, older divergence, dated at 47.8 million years, marks the first major internal diversification within *Liquorilactobacillus*, giving rise to two distinct evolutionary clades. One of these clades was composed predominantly of Usp proteins lacking the ATP-binding motif (Figure S1). The presence of these ATP-independent Usp variants in multiple *Liquorilactobacillus* species suggests an early functional divergence within the genus. The widespread distribution of these sequences implies that the ancestral stage of *Liquorilactobacillus* Usp proteins lacked the ATP-binding motif, which was subsequently retained in several lineages.

### Evolutionary history of *Usp* in *Liqourilacbacillus*

**Fig. 5:**
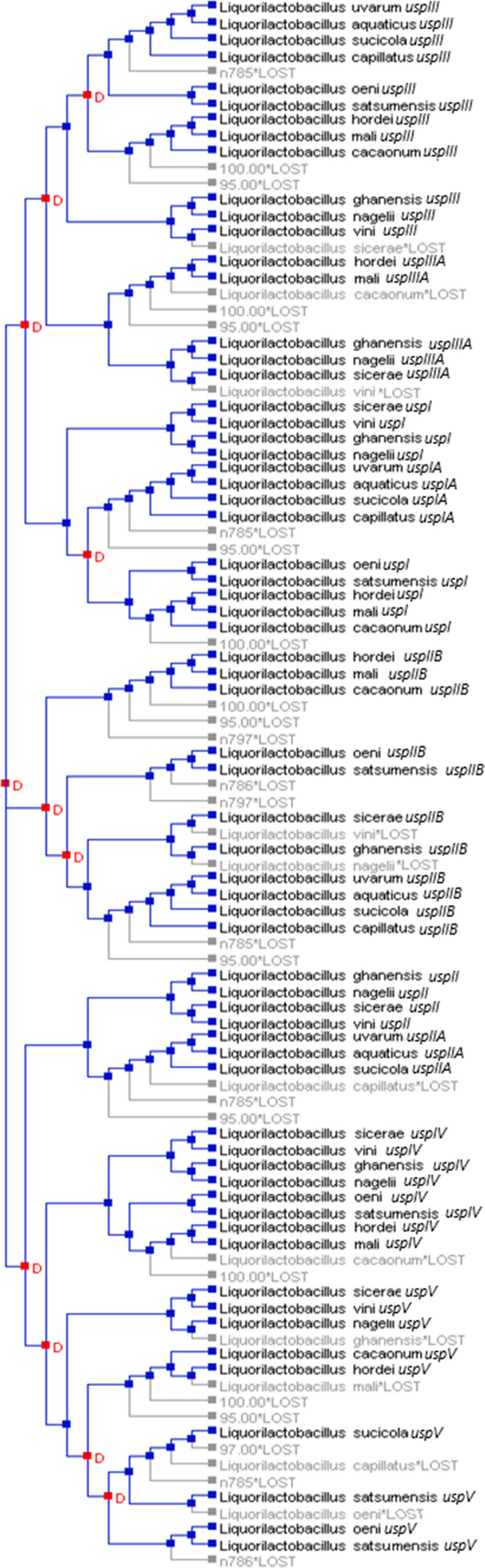
Phylogenetic reconstruction of gene gain, loss, and transfer events in *Liquorilactobacillus*. Phylogenetic tree showing the evolutionary history of gene families across *Liquorilactobacillus* species. Red squares (D) represent gene duplication events, and grey squares (*G*LOST) correspond to inferred gene losses. Bootstrap support values are shown at key nodes

**Fig. 6:**
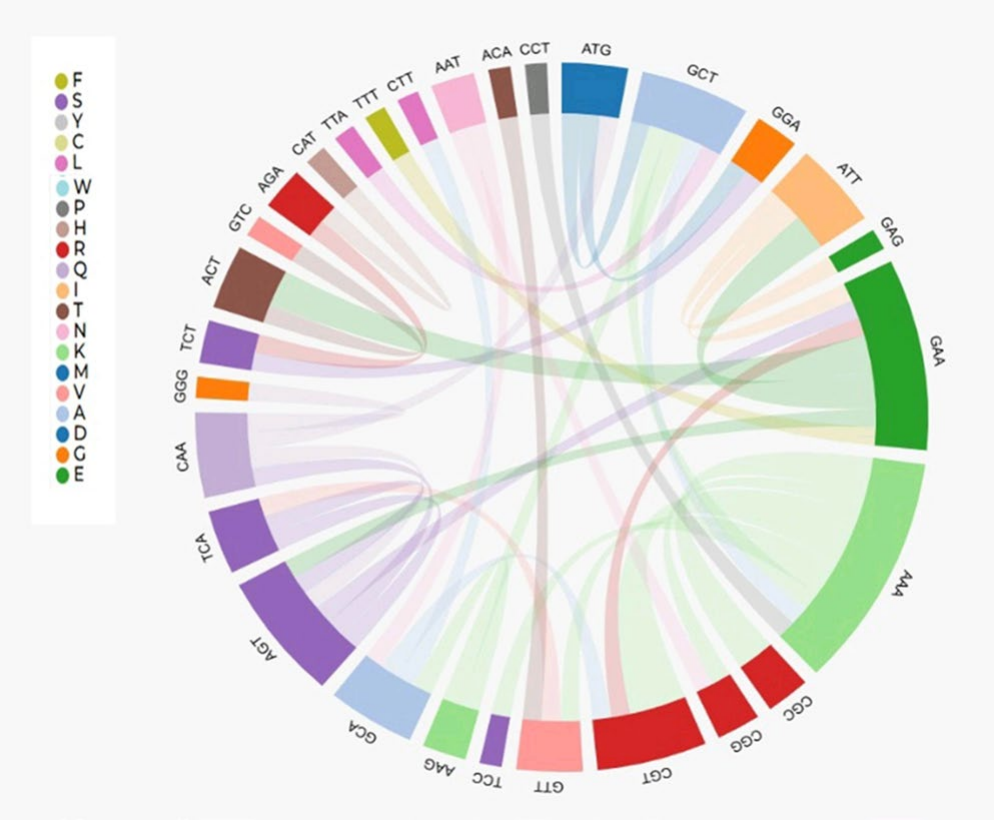
Codon substitution patterns in universal stress proteins (Usp) of *Liquorilactobacillus*. Chord diagram illustrating codon substitutions across *Liquorilactobacillus* species. Each colored segment represents a codon position in Usp proteins, and the ribbons connect codons that underwent AA changing substitutions. The width of the ribbons is proportional to the frequency of substitutions, highlighting conserved and variable positions within these stress-related proteins

**Fig. 7:**
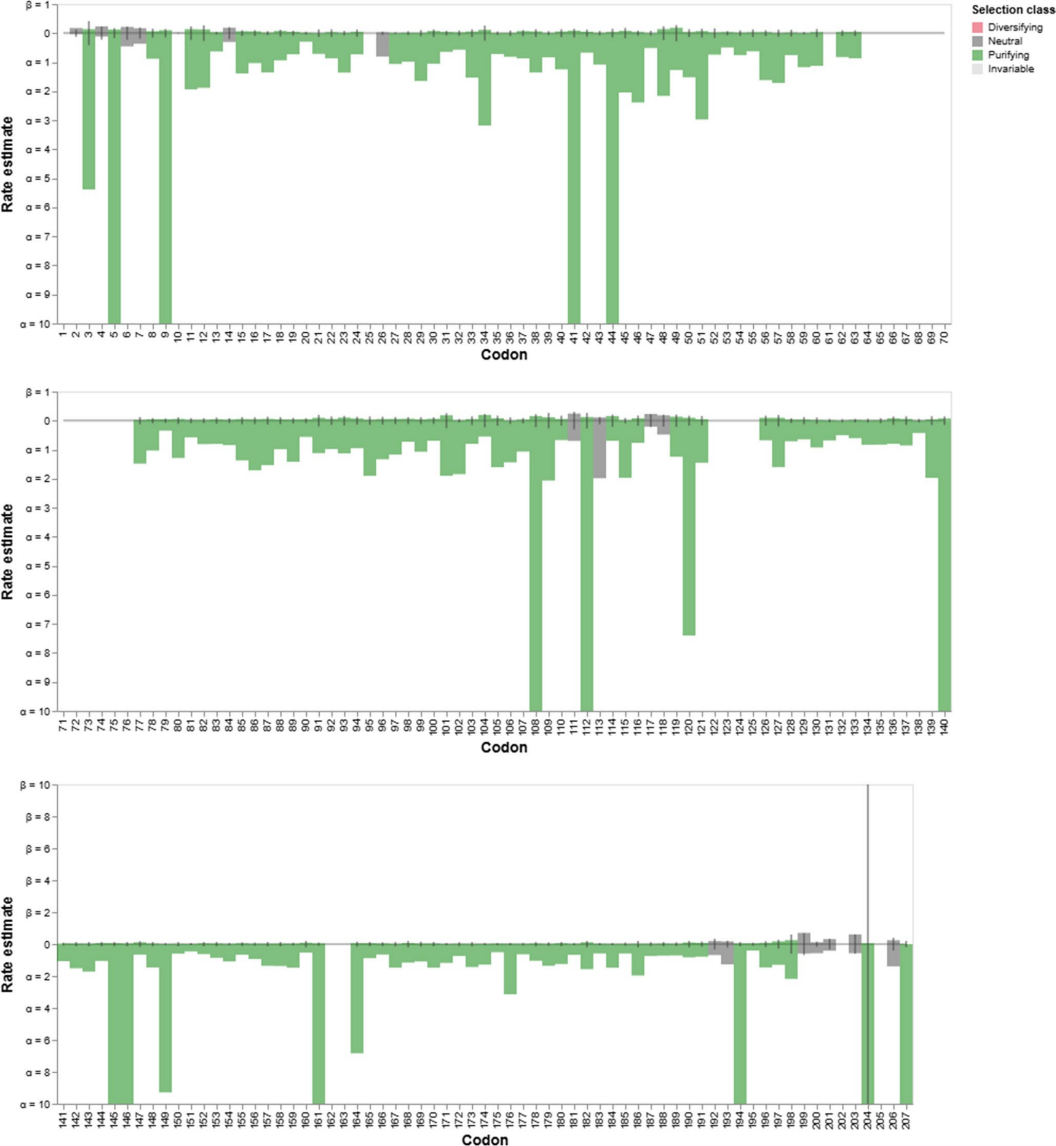
Codon-level selection analysis across the universal stress protein in *Liquorilactobacillus*. Site-by-site selection profiles obtained using codon-based models. Each bar represents the estimated substitution rate at a given codon position. Colors indicate the inferred selection class: diversifying (red), neutral (gray), purifying (green), and invariable (white)

The phylogenetic analyses of gene (Fig. 2) and protein (Fig. 4) revealed that Usp in the same species did not align together, indicating that the diversity of genes in all species of the genus originated from duplication events in their common ancestors. This hypothesis was confirmed by the reconciliation tree using the 13 *Liquorilactobacillus* species. In total, 12 events of gene duplication and 10 events of gene loss were detected (Fig. 5). There was the loss of one *usp* gene in *Li. ghanensis*, *Li. mali*, *Li. oeni*, *Li. nagelii* and *Li. sicerae* and two *usp* genes by *Li. vini*, *Li. capillatus* and *Li. cacaonum*. It is important to note that all species of the most basal clade 1 have lost at least one *usp* gene during the evolutionary process (Fig. 5). The duplication events that occurred in the evolutionary history resulted in the current diversity of the Usp proteins in this group of species. The dN/dS calculations for each USP codon showed 111 codons that were under the pattern of purifying selection, while 10 codons were neutral for this trait (Fig. 6). Besides, 19 codons showed no change. No activating selection points were found (Fig. 6). Additionally, Multi-hit codon substitution analysis revealed a total of 45 substitutions were mapped in the *Liquorilactobacillus* Usp (Fig. 7). Serine and alanine were the amino acids most frequently substituted by other amino acids, with four and eight substitutions, respectively. Interestingly, serine (seven substitutions) and alanine (six substitutions) were the amino acids that most frequently replaced other amino acids throughout the entire Usp protein. These substitutions were classified in three categories. The first category was composed of 15 nonsynonymous substitutions that preserved the chemical properties of the AA, such as six lysine-to-arginine substitutions, both basic AA. The second category was composed of 28 substitutions that resulted in changes to AA of different chemical groups. Examples include three lysine-to-alanine substitutions, reflecting a shift from a basic to a nonpolar residue. The third category represented synonymous replacements.

## Discussion

Universal stress proteins (Usp) occur in multiple copies across many bacterial species and often display a high degree of conservation. This family comprises from relatively small proteins of 14–15 kDa, when solely composed of one conserved Usp domain of approximately 100 AA, to larger proteins of 30 kDa when containing two *in tandem* Usp domains or the Usp domain fused to other functional regions or catalytic domains such as kinases, ion exchangers and permeases (Luo et al. 2023; Nabi et al. 2024). The Usp domain adopts a β-barrel fold that mediates interactions with diverse cellular partners (Sousa and MacKay 2001). The proteins analyzed here exhibited the conventional β-barrel fold with a single USP domain. This feature contrasts to proteins such as UspE from *Escherichia coli* that contains E1 and E2 *in tandem* USP domains (Kvint et al. 2003). Moreover, the data indicate that *Liquorilactobacillus* Usp are cytoplasmic, consistent with roles in responding to diverse stresses and in sensing cellular metabolic state. This information is also important because the *E*. *coli* UspB protein was initially classified as a universal stress protein, but this classification was revised after it was found to be a membrane protein (Tkaczuk et al. 2013).

The number of Usp paralogs per species of this genus, from three to six (Fig. 1), did not correlate significantly with genome size, suggesting that copy number is more closely related to ecological adaptation during speciation and to responses to specific stressors. Supporting this, the reconciled gene tree of 65 *usp* genes (Fig. 5) revealed multiple ancestral duplication and loss events. Duplications appear to have originated at ancestral nodes of clades rather than by recent duplications within single species (Figs. 2 and 5). Gene losses observed in some *Liquorilactobacillus* lineages may reflect functional redundancy from which some copies became dispensable following niche shifts after speciation. In addition, point mutations have shaped USP evolution. Amino acid substitutions were frequent in the evolution of this group of proteins to the point that a particular configuration produced an ATP-binding (Walker A/P-loop) domain (Figs. 3 and 4).

In this type of analysis, the natural impulse seems to be to focus on the sequence that exhibits a known motif and, consequently, to regard alterations in the amino acid sequence as a kind of degeneration of that functional signature. Thus, most analyses have traditionally been conducted from the perspective of functional loss. For instance, Aravind et al. (2002) defined in their work that UspFG-type proteins were evolutionarily older than UspA-type proteins, which lack the ATP-binding motif, and that the latter underwent more evolutionary events associated with the loss of ATP-binding function. In the present study, we propose to challenge this perception based on phylogenetic and evolutionary data. This was mainly because *Bacilli* species possess two Usp proteins: one containing the ATP-binding motif and another that either lacks or has a degenerated version of this signature. In the phylogenetic tree, the Usp protein that lacks the ATP-binding motif appears more basal in *Bacillus* and clustered with the Usp of *Liquorilactobacillus* that do not present the canonical motif. Based on evolutionary divergence analyses expressed in millions of years, we identified that the earliest Usp of *Liquorilactobacillus* emerged without the ATP-binding motif characterized by the signatures G-2X-T-(13/11X)-G-S. Subsequently, based on the accumulation of synonymous and non-synonymous substitutions, we detected the establishment of the G-X-TG-9X-G-S signature, creating an ATP-binding motif with a gain of function in the C-terminal region of approximately half of the Usp proteins of the genus. The second point is that the more central positions within the phylogeny of *Liquorilactobacillus* Usps also lack this motif. At this stage, it is essential to emphasize the evolutionary continuity of this substitution process, which generates a set of proteins with alterations in this signature, resulting in functional loss. This loss of the motif differed from that observed in the basal Usp of the phylogenetic tree, in which the basal signature G-2X-T-(13/11X)-G-S was represented at that point by the atavic signature S-X-TG-9X-G-A. In this case, we consider the use of the expression “degenerated ATP-binding motif” to be appropriate.

Substitutions in the Walker A/P-loop can alter structure sufficiently to preclude nucleotide binding. In *Escherichia coli*, class I Usp lacking the canonical ATP-binding motif and class II that retaining it perform distinct roles: class I proteins are implicated in resistance to oxidative stress, nutrient starvation (carbon, nitrogen, phosphate, sulfur), heavy-metal tolerance and iron scavenging, whereas class II Usp contribute to adhesion, motility (including swimming) and oxidative stress protection (Nabi et al. 2024). Even among *Liquorilactobacillus* Usp that preserve the A-loop, some lack the conserved lysine residue, which may impair nucleotide binding. That lysine and neighboring residues help position the nucleotide and stabilize the negative charge on the γ-phosphate, while the serine/threonine at the motif terminus contributes to Mg²⁺ coordination needed for that binding. Sequence diversity is concentrated in the inter-strand/loop regions of the USP fold. Both synonymous and non-synonymous substitutions were detected in the course of Usp evolution in *Liquorilactobacillus*. Many non-conservative substitutions occur at apparently resilient positions that do not compromise the overall fold. Overall, Usp proteins in *Liquorilactobacillus* were subject to strong purifying selection, preserving their essential function while allowing limited and localized variation. Codon-level analyses indicated that most sites are under purifying selection, with a subset of neutral and invariant sites and no evidence for pervasive positive selection (Fig. 6).

The Usp of *Liquorilactobacillus vini* are particularly informative for understanding stress responses in industrial contexts. Mendonça et al. (2020) reported expression profiles of the five *usp* genes in *Li. vini* under several forms of stress: ethanol, pH shifts, osmotic, oxidative and thermal stresses. These showed different levels of expression in different stress. The genes *uspI* (25x), uspII (48x), uspIV (25x) and uspV (3x) was highly induced in the presence of NaCl. On the other hand, *uspIII* was over-expressed in the presence of lactic acid (10x). In agreement with our phylogenetic results, the *usp*II was the most divergent *Li. vini* (Fig. 1) and it was the only gene upregulated by all tested stressors (Mendonça et al. 2020). Other *usp* genes exhibited stress-specific co-expression patterns, consistent with functional specialization among paralogs, but in a way that reflected their phylogenetic arrangement of protein clusters: *uspI* together with *uspIII* and *uspIV* together with *uspV*. It is worth noting that this clusterization coincided with the fact that these proteins may or may not present, respectively, the ATP-binding motif.

The number of universal stress proteins (USPs) varied among *Liquorilactobacillus* clades. Clade 1, comprising four species, exhibited the highest total number of USP proteins (*n* = 23), whereas clade 2, with only two species, encoded 11 USPs. When normalized by species number, clades 1 and 2 showed the highest USP density (5.75 and 5.5 USPs per species, respectively), while clade 4 presented the lowest average (4.0 USPs per species). Clade 3, composed of three species, encoded a total of 15 universal stress proteins, corresponding to an average of 5.0 USPs per species, an intermediate profile compared to the other clades (Fig. 2).

The interspecific variation observed among the 13 species of *Liquorilactobacillus* suggests a potential correlation between the number and properties of USPs and niche specialization within the genus, even though direct ecological fitness was not assessed. Although clades 1 and 4 each comprise four species, clade 1 encodes 23 USPs in total, while clade 4 encodes only 16 (Fig. 2). This discrepancy is unlikely to be a result of sampling bias, suggesting instead a clade-specific adaptation shaped by environmental pressures. Except for *Liquorilactobacillus ghanensis*, species in clade 1 are predominantly isolated from environments characterized by high ethanol concentrations, where chronic exposure to ethanol imposes multiple stressors, including protein denaturation, membrane damage, osmotic imbalance, and oxidative stress (Mendonça et al. 2020). In contrast, species in clade 4 are primarily isolated from more heterogeneous and less extreme environments, such as water (*Li. aquaticus*), fruits (*Li. uvarum*), plant sap (*Li. sucicola*), and tofu (*Li. capillatus*). These habitats impose stressors that are likely to be more transient and specific to each environment, rather than continuous exposure to stress, which could explain the smaller number of USPs in this clade (Bulgarelli et al. 2013; Yang et al. 2017).

In addition to the quantitative differences in USP number, clade-specific variation in USP structure was also observed. Clade 1 not only encodes more USPs overall (23) but also shows greater structural diversity, with 11 (47.8%) of these USPs lacking the ATP-binding domain. In contrast, only four (25%) of the USPs in clade 4 lack this domain. For instance, ATP-independent USPs such as those in the UspA family are known to protect cells from oxidative damage (Kvint et al. 2003). Additionally, it is worth highlighting that clade 2, composed of *Li. satsumensis* and *Li. oeni*, like clade 1, was also isolated from alcoholic fermentation environments. Despite comprising only two species, this clade collectively encodes 11 universal stress protein (USP) genes, of which five (45%) correspond to ATP-binding–deficient USPs, the same proportion observed in clade 1. Clade 3, formed by three species (*Li. hordei*, *Li. mali*, and *Li. cacaonum*), includes only *Li. cacaonum* as a non–alcoholic beverage isolate, having been recovered from cacao. Nevertheless, this clade encodes 15 USP genes, six of which (40%) lack the ATP-binding motif.

These findings underscore that the differences in both the quantity and the functional diversity of USPs between *Liquorilactobacillus* clades are likely reflective of distinct evolutionary pressures driven by their respective ecological niches.

The genes encoding universal stress proteins (Usp) in *Liquorilactobacillus* provide insight into the genetic repertoire associated with adaptation to fermentative and industrial environments. Although the precise functions of these proteins remain largely unknown, the organization and composition of *usp* genes across different species suggest that they may have played an important evolutionary role in adaptation to such niches. These observations highlight the relevance of exploring the genomic variability of *usp*, paving the way for a better understanding of how *Liquorilactobacillus* maintains resilience under environmental stress.

## Conclusion

The evolutionary history of universal stress proteins in *Liquorilactobacillus* reveals a balance between conservation and diversification. While strong purifying selection maintains the structural and functional integrity of the USP domain, gene duplication, gene loss, and occasional AA substitutions have provided opportunities for functional specialization across species and lineages. These patterns highlight the role of Usp as a central element in stress response and ecological adaptation. Future functional and structural studies will be essential to clarify how specific paralogs contribute to survival under diverse environmental and industrial conditions.

## Supplementary Information

Below is the link to the electronic supplementary material.

Supplementary figure 1 (PNG 1.01 MB)

High Resolution Image (TIF 275 KB)

ESM 2 (XLSX 55.4 KB)

ESM 3 (DOCX 25.7 KB)

## Data Availability

No datasets were generated or analysed during the current study.
