# Supplementary figures and images for: Diversity and evolutionary dynamics of universal stress proteins in the Liquorilactobacillus genus
Source: World J Microbiol Biotechnol. 2026 Feb 28;42(3):111. doi: 10.1007/s11274-026-04821-4 (PMC12950057; doi:10.1007/s11274-026-04821-4)

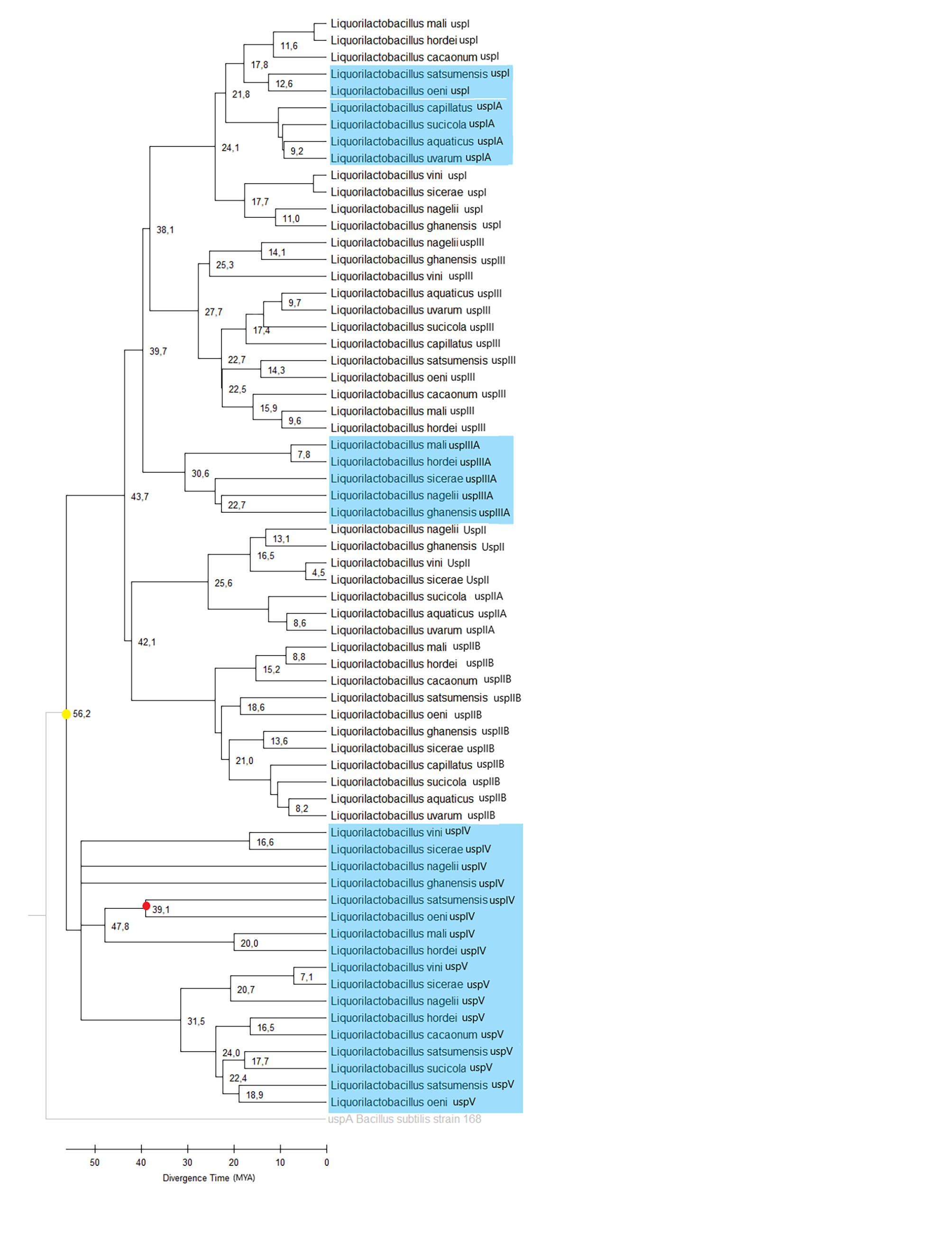

Supplement: Supplementary file 1 — (PNG 1.01 MB) [file 11274_2026_4821_Fig8_ESM.png]

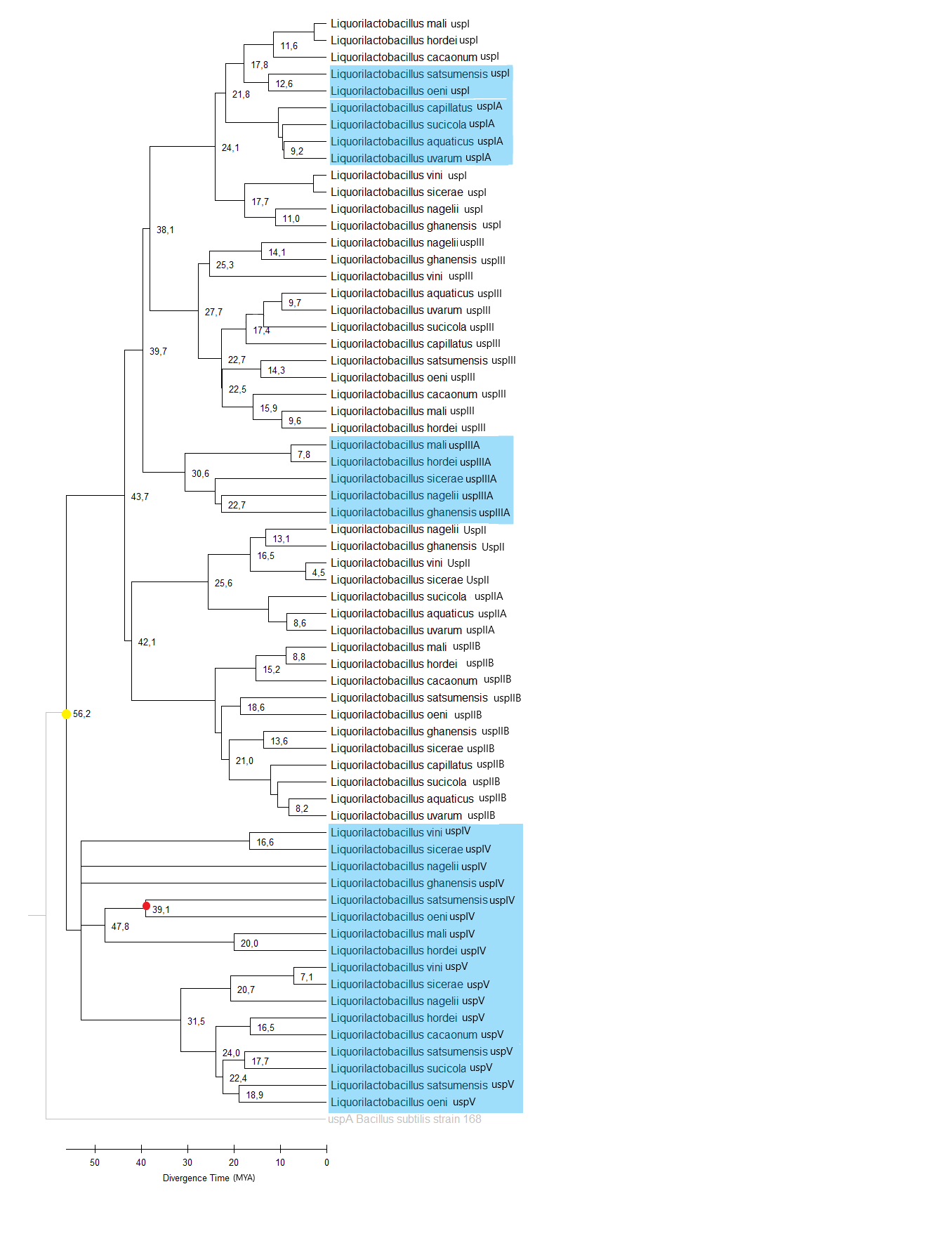

Supplement: Supplementary file 2 — High Resolution Image (TIF 275 KB) [file 11274_2026_4821_MOESM1_ESM.tif]
