# Supplementary material for: Diversity and evolutionary dynamics of universal stress proteins in the Liquorilactobacillus genus
Source: World J Microbiol Biotechnol. 2026 Feb 28;42(3):111. doi: 10.1007/s11274-026-04821-4 (PMC12950057; doi:10.1007/s11274-026-04821-4)
Supplement: Supplementary file 4 — (DOCX 25.7 KB) [file 11274_2026_4821_MOESM3_ESM.docx]

**Diversity and Evolutionary Dynamics of Universal Stress Proteins in the *Liquorilactobacillus* Genus**

**Santos et al.**

**Table S1.** Universal Stress Proteins in the *Liquorilactobacillus* genus as recovered from the GenBank/NCBI database and their reclassification according to homology to *Li. vini* proteins.

| **Species** | **Original name** | **Acession** | **size (aa)** | **Name in this study** |
| --- | --- | --- | --- | --- |
| Liquorilactobacillus aquaticus DSM 21051 | universal stress protein UspA | KRM97543.1 | 167 | UspIIB |
| Liquorilactobacillus aquaticus DSM 21051 | universal stress protein UspA | KRM96309.1 | 142 | UspIIA |
| Liquorilactobacillus aquaticus DSM 21051 | universal stress protein Usp | KRM96673.1 | 148 | UspIA |
| Liquorilactobacillus aquaticus DSM 21051 | universal stress protein Usp | KRM96113.1 | 154 | UspIII |
| Liquorilactobacillus cacaonum DSM 21116 | universal stress protein | KRM92658.1 | 152 | UspIII |
| Liquorilactobacillus cacaonum DSM 21116 | universal stress protein UspA-like nucleotide-binding protein | KRM92118.1 | 142 | UspV |
| Liquorilactobacillus cacaonum DSM 21116 | universal stress protein | KRM90860.1 | 156 | UspIIB |
| Liquorilactobacillus cacaonum DSM 21116 | universal stress protein | KRM90343.1 | 148 | UspI |
| Liquorilactobacillus capillatus DSM 19910 | universal stress protein | KRL03149.1 | 139 | UspIA |
| Liquorilactobacillus capillatus DSM 19910 | universal stress protein | KRL02451.1 | 169 | UspIII |
| Liquorilactobacillus capillatus DSM 19910 | universal stress protein UspA | KRL00103.1 | 164 | UspIIB |
| Liquorilactobacillus sucicola DSM 21376 = JCM 15457 | universal stress protein | KRN06588.1 | 155 | UspIII |
| Liquorilactobacillus sucicola DSM 21376 = JCM 15457 | universal stress protein UspA-like nucleotide-binding protein | KRN06646.1 | 144 | UspV |
| Liquorilactobacillus sucicola DSM 21376 = JCM 15457 | universal stress protein UspA | KRN06157.1 | 164 | UspIIB |
| Liquorilactobacillus sucicola DSM 21376 = JCM 15457 | universal stress protein | KRN05120.1 | 151 | UspIA |
| Liquorilactobacillus sucicola DSM 21376 = JCM 15457 | universal stress protein UspA | KRN05470.1 | 142 | UspIIA |
| Liquorilactobacillus mali KCTC 3596 = DSM 20444 | universal stress protein UspA family protein | EJF01455.1 | 161 | UspIIIA |
| Liquorilactobacillus mali KCTC 3596 = DSM 20444 | universal stress protein | EJF00941.1 | 149 | UspIII |
| Liquorilactobacillus mali KCTC 3596 = DSM 20444 | universal stress protein | EJF00070.1 | 155 | UspIV |
| Liquorilactobacillus mali KCTC 3596 = DSM 20444 | universal stress protein UspA-like nucleotide-binding protein | EJE99833.1 | 152 | UspIIB |
| Liquorilactobacillus mali KCTC 3596 = DSM 20444 | universal stress protein | EJE98245.1 | 131 | UspI |
| Liquorilactobacillus hordei DSM 19519 | universal stress protein | KRL07798.1 | 155 | UspIIIA |
| Liquorilactobacillus hordei DSM 19519 | universal stress protein | KRL07441.1 | 156 | UspIII |
| Liquorilactobacillus hordei DSM 19519 | universal stress protein UspA-like nucleotide-binding protein | KRL07182.1 | 142 | UspIV |
| Liquorilactobacillus hordei DSM 19519 | universal stress protein | KRL07039.1 | 186 | UspIIB |
| Liquorilactobacillus hordei DSM 19519 | universal stress protein UspA family protein | KRL06285.1 | 161 | UspI |
| Liquorilactobacillus hordei DSM 19519 | universal stress protein | KRL06032.1 | 155 | UspV |
| Liquorilactobacillus uvarum DSM 19971 | universal stress protein UspA | KRL39102.1 | 175 | UspIA |
| Liquorilactobacillus uvarum DSM 19971 | universal stress protein | KRL38179.1 | 156 | UspIIB |
| Liquorilactobacillus uvarum DSM 19971 | universal stress protein UspA | KRL37206.1 | 142 | UspIII |
| Liquorilactobacillus uvarum DSM 19971 | universal stress protein | KRL36771.1 | 148 | UspIIA |
| Liquorilactobacillus satsumensis DSM 16230 = JCM 12392 | universal stress protein UspA family protein | KRL98105.1 | 163 | UspIII |
| Liquorilactobacillus satsumensis DSM 16230 = JCM 12392 | universal stress protein UspA | KRL97977.1 | 159 | UspV |
| Liquorilactobacillus satsumensis DSM 16230 = JCM 12392 | universal stress protein UspA-like nucleotide-binding protein | KRL97789.1 | 142 | UspI |
| Liquorilactobacillus satsumensis DSM 16230 = JCM 12392 | universal stress protein | KRL97549.1 | 159 | UspV- |
| Liquorilactobacillus satsumensis DSM 16230 = JCM 12392 | universal stress protein | KRL96731.1 | 160 | UspIIB |
| Liquorilactobacillus satsumensis DSM 16230 = JCM 12392 | universal stress protein UspA-like nucleotide-binding protein | KRL96752.1 | 144 | UspIV |
| Liquorilactobacillus oeni DSM 19972 | universal stress protein UspA-like nucleotide-binding protein | KRL05697.1 | 143 | UspIII |
| Liquorilactobacillus oeni DSM 19972 | universal stress protein | KRL05783.1 | 160 | UspV |
| Liquorilactobacillus oeni DSM 19972 | universal stress protein | KRL04945.1 | 150 | UspI |
| Liquorilactobacillus oeni DSM 19972 | universal stress protein UspA | KRL04187.1 | 161 | UspIIB |
| Liquorilactobacillus oeni DSM 19972 | universal stress protein UspA family protein | KRL03942.1 | 161 | UspIV |
| Liquorilactobacillus mali KCTC 3596 = DSM 20444 | universal stress protein | KRN11533.1 | 149 | UspIIIA |
| Liquorilactobacillus mali KCTC 3596 = DSM 20444 | universal stress protein | KRN10479.1 | 131 | UspIII |
| Liquorilactobacillus mali KCTC 3596 = DSM 20444 | universal stress protein UspA-like nucleotide-binding protein | KRN10318.1 | 148 | UspIV |
| Liquorilactobacillus mali KCTC 3596 = DSM 20444 | universal stress protein UspA | KRN10086.1 | 167 | UspIIB |
| Liquorilactobacillus mali KCTC 3596 = DSM 20444 | universal stress protein | KRN09164.1 | 155 | UspI |
| Liquorilactobacillus mali KCTC 3596 = DSM 20444 | universal stress protein | KRN09028.1 | 155 | UspIII |
| Liquorilactobacillus nagelii DSM 13675 | universal stress protein | QYH54943.1 | 150 | UspII |
| Liquorilactobacillus nagelii DSM 13675 | universal stress protein | QYH55419.1 | 142 | UspV |
| Liquorilactobacillus nagelii DSM 13675 | universal stress protein | KRL40231.1 | 143 | UspIV |
| Liquorilactobacillus nagelii DSM 13675 | universal stress protein | KRL39961.1 | 149 | UspIIIA |
| Liquorilactobacillus nagelii DSM 13675 | universal stress protein | KRL39963.1 | 150 | UspI |
| Liquorilactobacillus vini JP7.8.9 | UspI | a | 150 | UspI |
| Liquorilactobacillus vini JP7.8.9 | uspII | b | 141 | uspII |
| Liquorilactobacillus vini JP7.8.9 | uspIII | c | 153 | uspIII |
| Liquorilactobacillus vini JP7.8.9 | uspIV | d | 165 | uspIV |
| Liquorilactobacillus vini JP7.8.9 | uspV | e | 143 | uspV |
| Liquorilactobacillus sicerae CUPV261 | universal stress protein | f | 143 | UspV |
| Liquorilactobacillus sicerae CUPV261 | universal stress protein | g | 141 | UspII |
| Liquorilactobacillus sicerae CUPV261 | universal stress protein | h | 165 | UspIV |
| Liquorilactobacillus sicerae CUPV261 | universal stress protein | i | 162 | UspIIB |
| Liquorilactobacillus sicerae CUPV261 | universal stress protein | j | 150 | UspIIIA |
| Liquorilactobacillus sicerae CUPV261 | universal stress protein | k | 150 | UspI |
| Liquorilactobacillus ghanensis DSM 18630 | universal stress protein | KRM04400.1 | 150 | UspI |
| Liquorilactobacillus ghanensis DSM 18630 | universal stress protein | WP_415617258.1 | 149 | UspIIIA |
| Liquorilactobacillus ghanensis DSM 18630 | universal stress protein | WP_057872316.1 | 141 | UspII |
| Liquorilactobacillus ghanensis DSM 18630 | universal stress protein | WP_415589409.1 | 150 | UspIII |
| Liquorilactobacillus ghanensis DSM 18630 | universal stress protein | WP_057870683.1 | 162 | UspIV |
| Liquorilactobacillus ghanensis DSM 18630 | universal stress protein UspA | WP_415608637.1 | 162 | UspIIB |

1. MTQYYQEILVPVDGSENAEHALKQAVQIAKRNNSRLEILNVIDIRNFTNAFGSMLDMNGDIVYSTFNSVESYLNKLKQAIKQQANFENVRIHARFGSPRVVIANDFLTDYTIDLIVMGKTGSNPVERFLTGSVTDYVTRNAKCDIIIIHK
2. MYKNILIGIDGSKQAKQAFEVGCNLAKVLSAKVSLLWVVNRDRSMDVSFGVGAEFYQDVADQAKEKIKPYQDAAKEKGVEVKAEVLIGNVKEVLSDTYPKEHQIDLIVIGQTGMNSIEKVVVGSHTSYVVRNSACDVLVVK
3. MINYQRILVPIDGSKGAKIALNKAIKIVKENQAHLDILKVMDMNSLDLGNTGLILDGEQVYQIEQANESYLTKLNQELVKKYDLSSKQFHVHLRFGNPKVVIVQDFQPEYHNDLIVVGSTGKNFLERLVMGSVASFVVREASCDVLLARSEK
4. MKKHDVDLNFELANRLFSEILVAVDEDDSESSLAAFKYALSMAKTNQATLGIVTVLELEDLNVFEALSPEKRTAIRQNLEAALKLYIAKAHEVGVEKVKAFIREGKPAATIINDVIPVFRPDVLICGSKTKPINNRQKIFIGSQASYLAQNAPCSVMVIRPSFNK
5. MEDDFKKILVGVDDSEDALLAFRYAMKRARATNAELIIVSVLESNEMSVYQALSKDYIHGEREDLEKHILKYQKLAQDAGVKKVRSIVAEGNAGEAIVKDVIPHVQPDLLIIGSYSKKGLARHFGSQAAYMAKYAPVSVLVIR*
6. MEDDFKKILVGVDDSEDALLAFRYAMKRARTTNAELIIVSVLESNEMSVYQALSRDYIHGEREDLEKHILKYQKLAQDAGVKKVRSVVAEGNAGEAIVKDVIPHVQPDLLIIGSYSKTGLARRFGSQAAYMAKYAPVSVLVIR
7. MYKNILIGIDGSKQAKQAFEVGCSLAKALSAKVSLLWVVNRDRSMDVSFGVGAEFYQDVADQAKEKIKPYQDAAKEKGIEVKAEVLIGNVKEVLSDTYPKEHQIDLIVIGQTGMNSIEKVVVGSHTSYVVRNSACDVLVVK
8. MKRHDVDLNFELVDRPFSQILVAVDEDDSDSSLAAFKYALSMAKTNRATLGIVTVLELEDLNVFEALSPEKRTAIRQNLEATLKLYIAKAHDVGIEKVKAFIREGKPAATIVNDVIPTFRPDVLICGSKTKSVNSRQKIFIGSQASYLAQNAPCSVMVIRPAFNN
9. MLQQYNNILVPIDGSKEAELAFKKAVAVTRRNGDSAKLHLLHVVDTRAFQNISSFDTAMVEQVTETAKKTLENYVAEAQKAGLKNVDYSIEYGAPKVIIAKETPADKDIDLIMIGATGLNAVERLLIGSVTEYVTRTAACDVLVVRTDLDNKPVLNEKKSKK
10. MNQQPYQRILLAHDGSKSAEKALDIAADYARENNIQLDILYVLDSSIVGFGNTHAAPSEDDLYLFEEKAISLLDRIKKRLVACGSSVYQVHVHLRFGDPRTIIAKEFPDEYRNDLIVLGSSTKNYLQRIFTGSVSSYVIRTAACDALIIR
11. MTQYYQEILAPVDGSKNAEHALKQAVQIAKRNNSRLEILNVIDIRNFTNAFGSMLDMNGDIVYSTFNSVESYLNKLKQAIKQQANFENVRIHARFGSPRVVIANDFLTDYAIDLIVMGKTGSNPVERFLTGSVTDYVTRNAKCDIIIIHK
